# Supplementary material for: Predicting visual function by interpreting a neuronal wiring diagram
Source: Nature. 2024 Oct 2;634(8032):113–23. doi: 10.1038/s41586-024-07953-5 (PMC11446822; doi:10.1038/s41586-024-07953-5)
Supplement: Supplementary file 6 — CRF and ERF predictions for individual TmY4 and TmY9 cells. Analogous to Supplementary Data 3, but for TmY target types. Shown are the top four monosynaptic pathways, the strongest pathway passing through each of the top ten intermediary types (ranking from Extended Data Fig. 7), and the trisynaptic pathway Tm1–TmY–Dm3–TmY (see the section entitled Prediction of spatial normalization). [file 41586_2024_7953_MOESM6_ESM.zip › DataS4/TmY9q/720575940618696016.pdf]

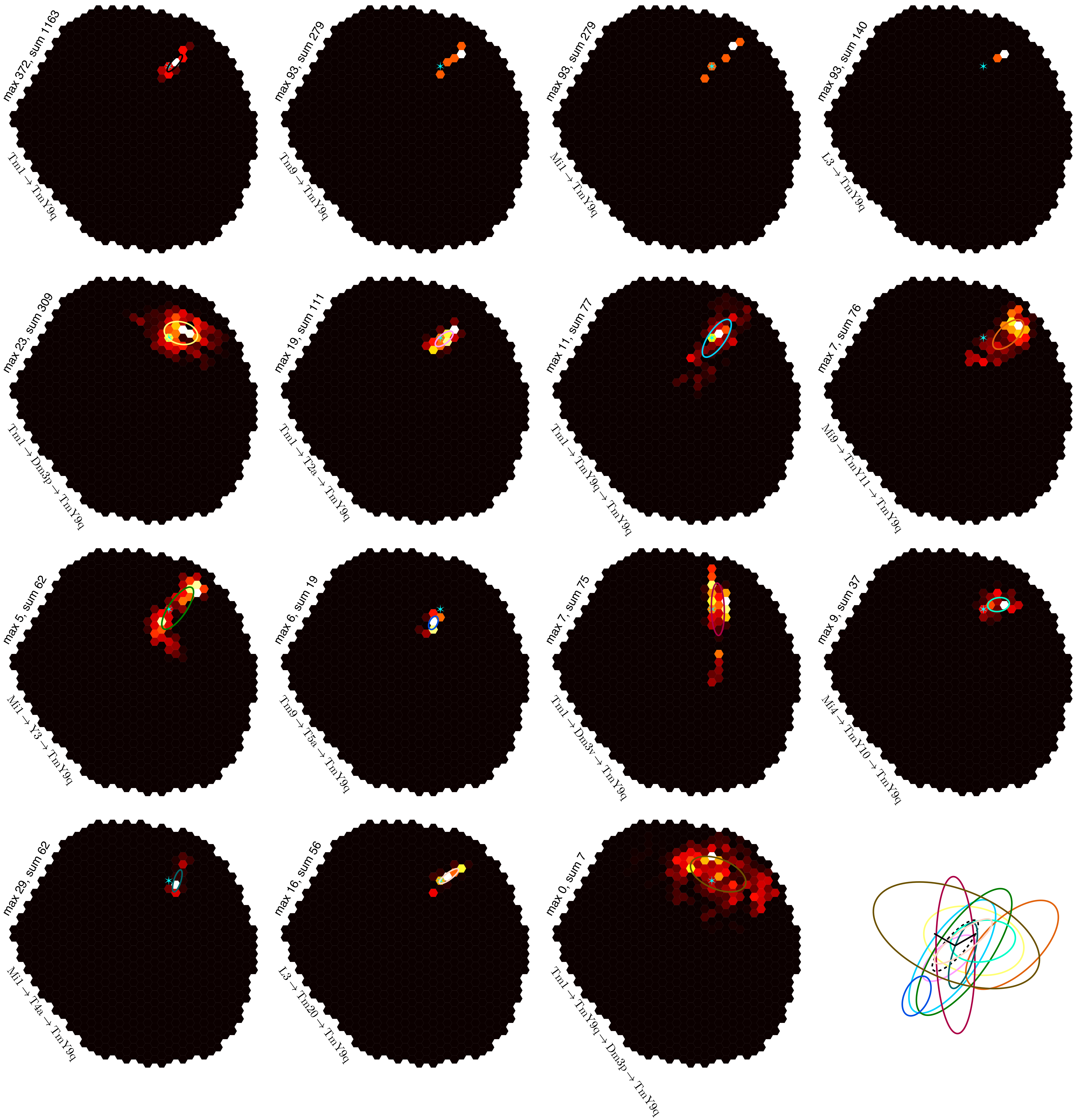

max 372, sum 1163
Tm1 → TmY9q
max 93, sum 279
Tm9 → TmY9q
max 93, sum 279
Mi1 → TmY9q
max 93, sum 140
L3 → TmY9q
max 23, sum 309
Tm1 → Dm3p → TmY9q
max 19, sum 111
Tm1 → T2a → TmY9q
max 11, sum 77
Tm1 → TmY9q → TmY9q
max 7, sum 76
Mi9 → TmY11 → TmY9q
max 5, sum 62
Mi1 → Y3 → TmY9q
max 6, sum 19
Tm9 → T5a → TmY9q
max 7, sum 75
Tm1 → Dm3v → TmY9q
max 9, sum 37
Mi4 → TmY10 → TmY9q
max 29, sum 62
Mi1 → T4a → TmY9q
max 16, sum 56
L3 → Tm20 → TmY9q
max 0, sum 7
Tm1 → TmY9q → Dm3p → TmY9q
